# Supplementary material for: Opsin Gene Duplication in Lepidoptera: Retrotransposition, Sex Linkage, and Gene Expression
Source: Mol Biol Evol. 2023 Nov 3;40(11):msad241. doi: 10.1093/molbev/msad241 (PMC10642689; doi:10.1093/molbev/msad241)
Supplement: msad241_Supplementary_Data [file msad241_supplementary_data.pdf]

Supplementary data - Opsin gene duplication in Lepidoptera: retrotransposition, sex linkage, and gene expression

Peter O. Mulhair[1,*], Liam Crowley[1], Douglas H. Boyes[2], Owen T. Lewis[1], and Peter W.H. Holland[1]

[1]Department of Biology, University of Oxford, 11a Mansfield Road, Oxford OX1 3SZ, UK
[2]UK Centre for Ecology Hydrology, Wallingford, OX10 8BB, UK
[*]Corresponding author: Peter O. Mulhair, peter.mulhair@biology.ox.ac.uk

Supplementary Figures

Supplementary Figure S1: Lepidoptera species tree and opsin copy number.
Supplementary Figure S2: Opsin gene tree.
Supplementary Figure S3: *Euclidia mi* and *Ochlodes sylvanus* Blue opsin tandem duplications.
Supplementary Figure S4: Gene tree and alignment of blue opsin duplicate genes.
Supplementary Figure S5: Autosome-Z chromosome fusion events in Tortricidae and *Anorthoa munda*.
Supplementary Figure S6: Noctuoidea LW opsin gene tree.
Supplementary Figure S7: Summary of LWS2 paralog genomic locations and duplication history.
Supplementary Figure S8: Genomic locations of LWS2 paralog in Erebidae species.
Supplementary Figure S9: TPM values for opsin genes in different lifestages of Vapourer moth (*Orgyia antiqua*).
Supplementary Figure S10: Opsin structure with retinal binding sites and overlap with posi- tively selected sites.

Supplementary Tables

Supplementary Table S1: List of species and their accession numbers used in this study.

Supplementary Figures

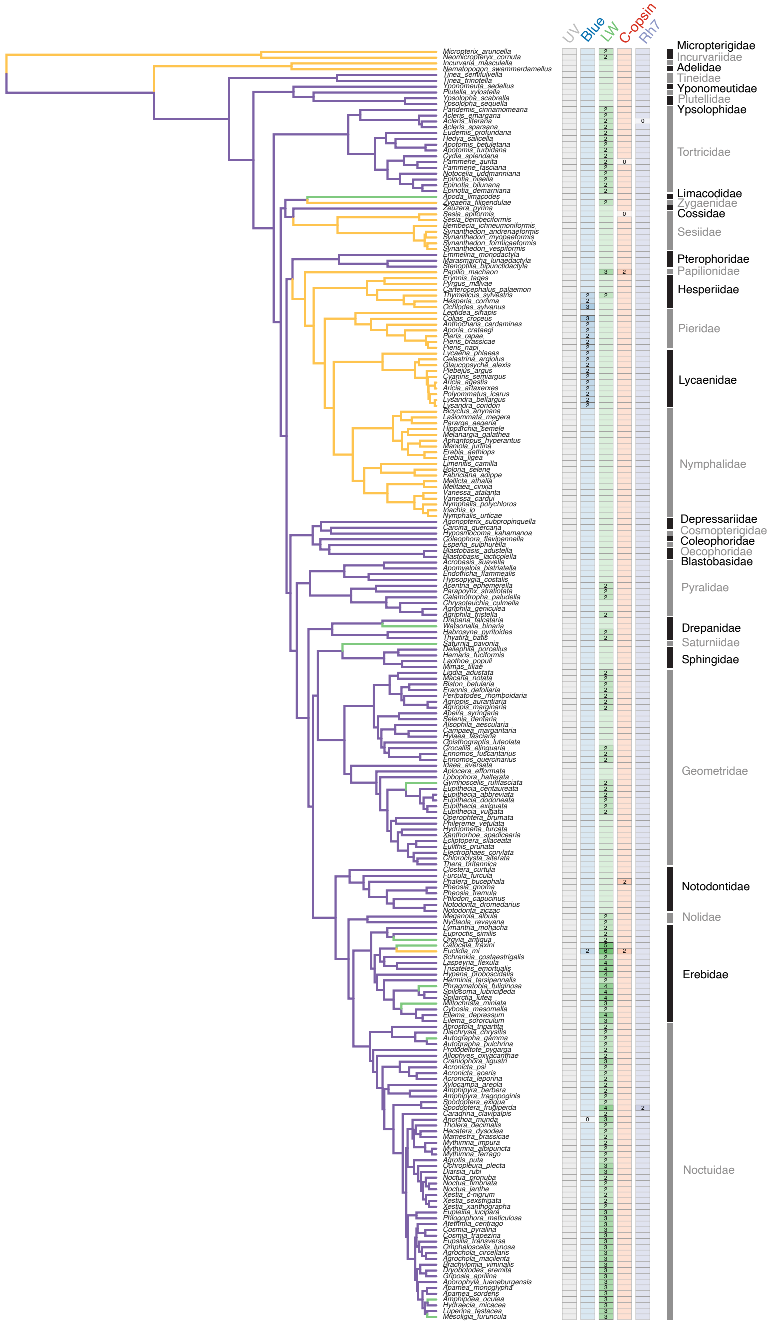

Supplemental Figure S1: Lepidoptera species tree and opsin copy number. Species tree on the left represents the Lepidoptera phylogeny inferred from BUSCO gene set of 1,281 genes, using a maximum likelihood supermatrix approach. Species names are given, and their corresponding branch colour denotes whether they are nocturnal (purple), diurnal (yellow), or have evidence for activity in both (green). Coloured matrix represents copy number for each opsin gene in each species. The colours correspond to those in the species tree in Figure 1A in the main text, numbers are added to the boxes to show the number of copies of the given opsin gene (shown for opsin genes that deviated from the normal single copy). The black and grey bars on the right show represent the lepidopteran families in the species tree.

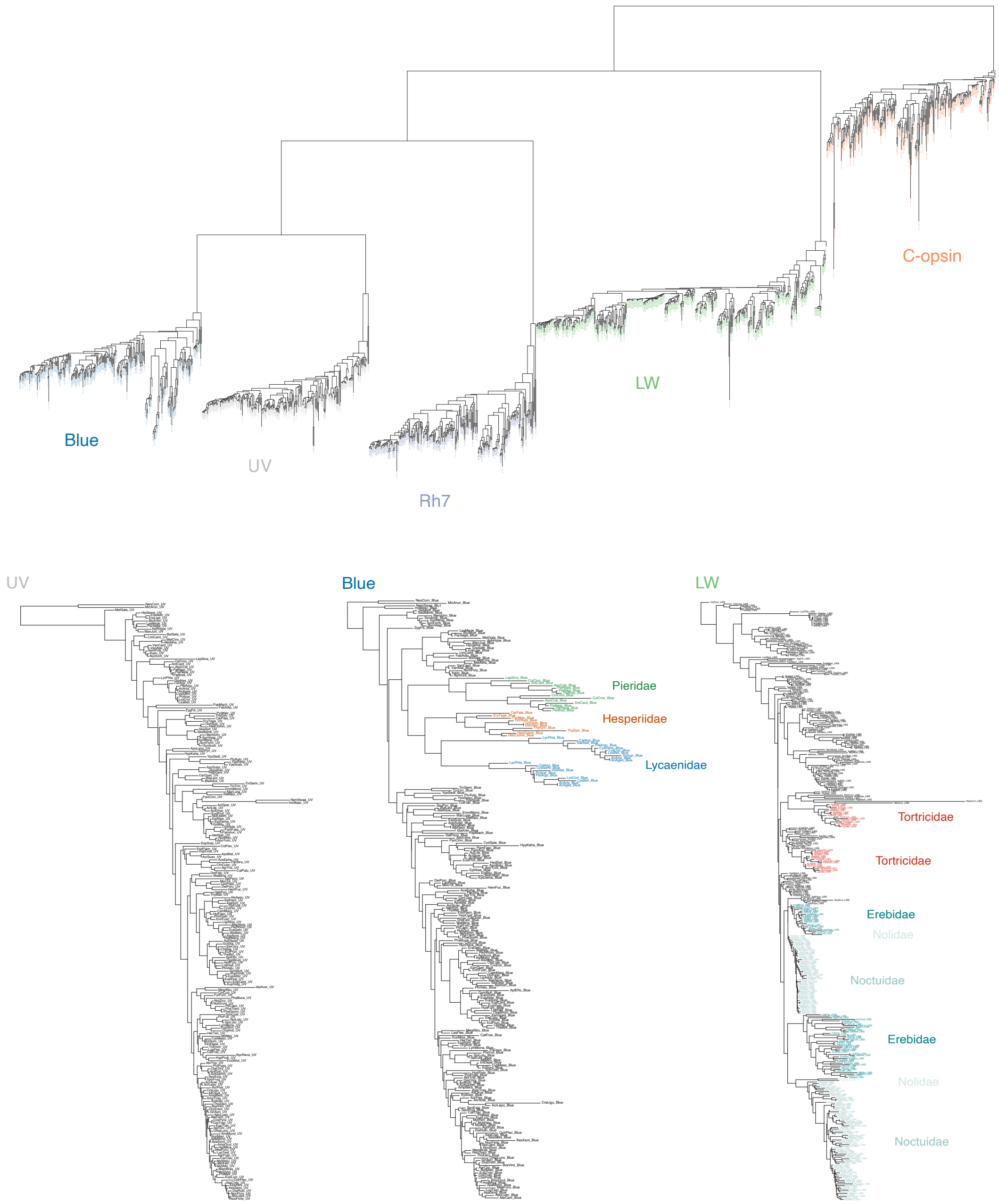

Supplemental Figure S2: Opsin gene trees. (Upper) Gene tree of all opsin sequences inferred using a maximum likelihood approach using IQtree. The tree and gene clade colours correspond to those in Figure 1B in the main text. (Lower) Gene trees for each of the visual opsins (UV, Blue, LW), with major duplications shared between a large number of species coloured and labelled with the lepidopteran family within which it occurred.

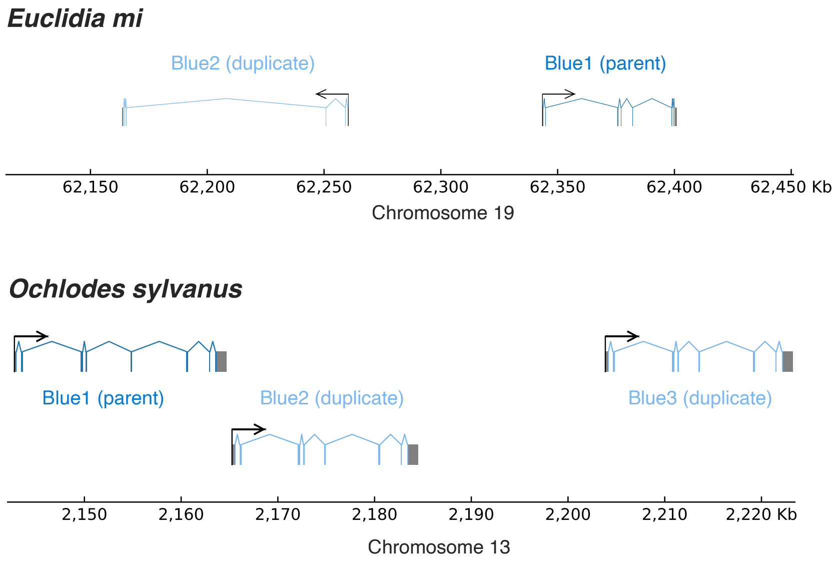

Supplemental Figure S3: *Euclidia mi* and *Ochlodes sylvanus* Blue opsin tandem duplication. Gene tracks showing independant tandem duplication of the Blue opsin gene in *Euclidia mi* (Mother Shipton moth) and *Ochlodes sylvanus* (Large skipper butterfly). Both blue copies are located on chromosome 19 in *Euclidia mi*, with the duplicated Blue opsin located 83kb upstream of the parent copy in the opposite orientation. In *Ochlodes sylvanus* three blue opsin copies present on chromosome 13 represent one duplication shared with other skipper butterflies (Blue2 in the figure) and another more recent duplication just found in *Ochlodes sylvanus* in our dataset (Blue3). All blue opsin copies are all in the same orientation and closely linked in the genome, with 600 base pairs and 19Kb of intergenic space between them. The more recent duplicate (Blue3) likely emerged as a result of duplication of the Blue2 copy, as they share identical sequence homology.

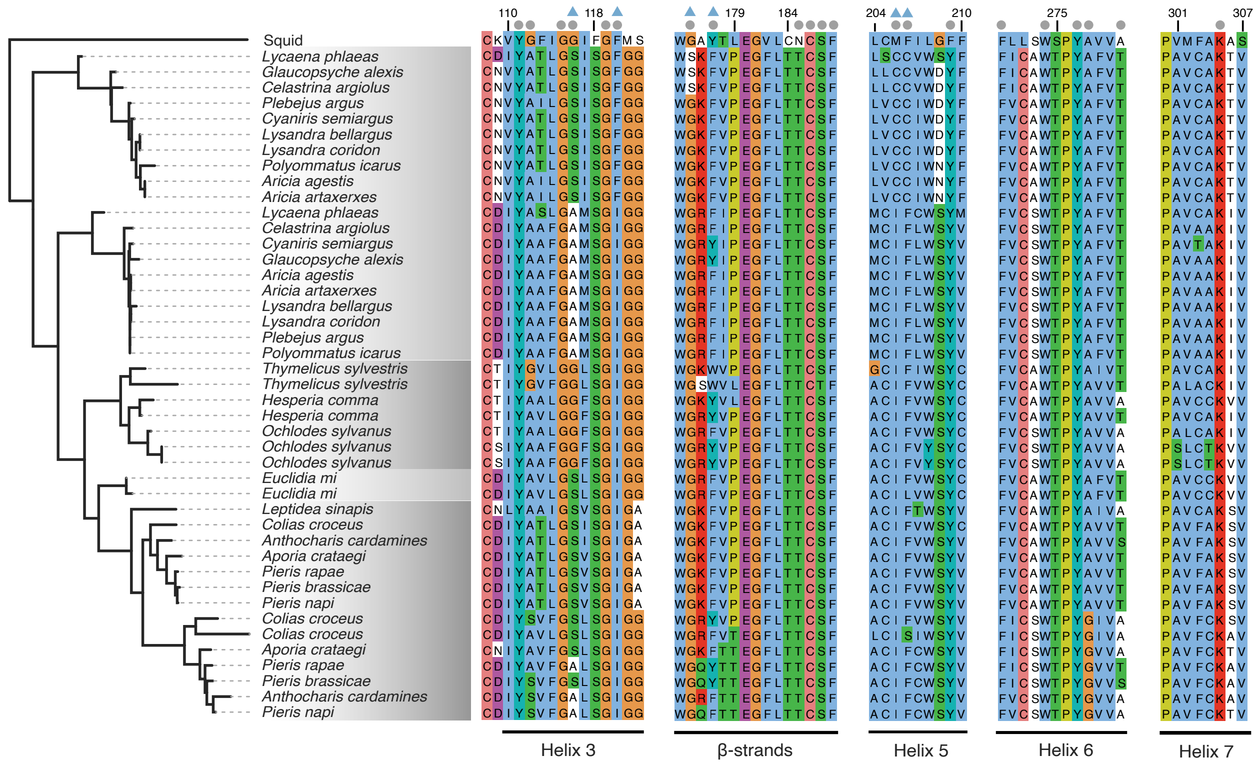

Supplemental Figure S4: Gene tree and alignment of blue opsin duplicate genes. Left; Maximum likelihood gene tree of blue opsin genes which underwent duplication in certain lepidopteran species. Alternating grey colours correspond to specific families within which the blue opsin gene underwent a duplication event (Lycaenidae, Hesperidae, Erebidae, and Pieridae). The Squid opsin (taken from *Todarodes pacificus*, accession no. CAA4990) is used as an outgroup. **Right;** Partial alignment of the corresponding blue opsin paralogs, showing regions of the opsin protein corresponding to helix loops and beta-strands. Dots and triangles above the alignment are obtained from (Liénard et al. 2021) and correspond to sites within 5 Å of any carbon atom in the retinal polyenechain, and sites shown to result in spectral shift of the blue opsin gene, respectively. The numbered sites above the alignment correspond to amino acid sites within the squid opsin protein.

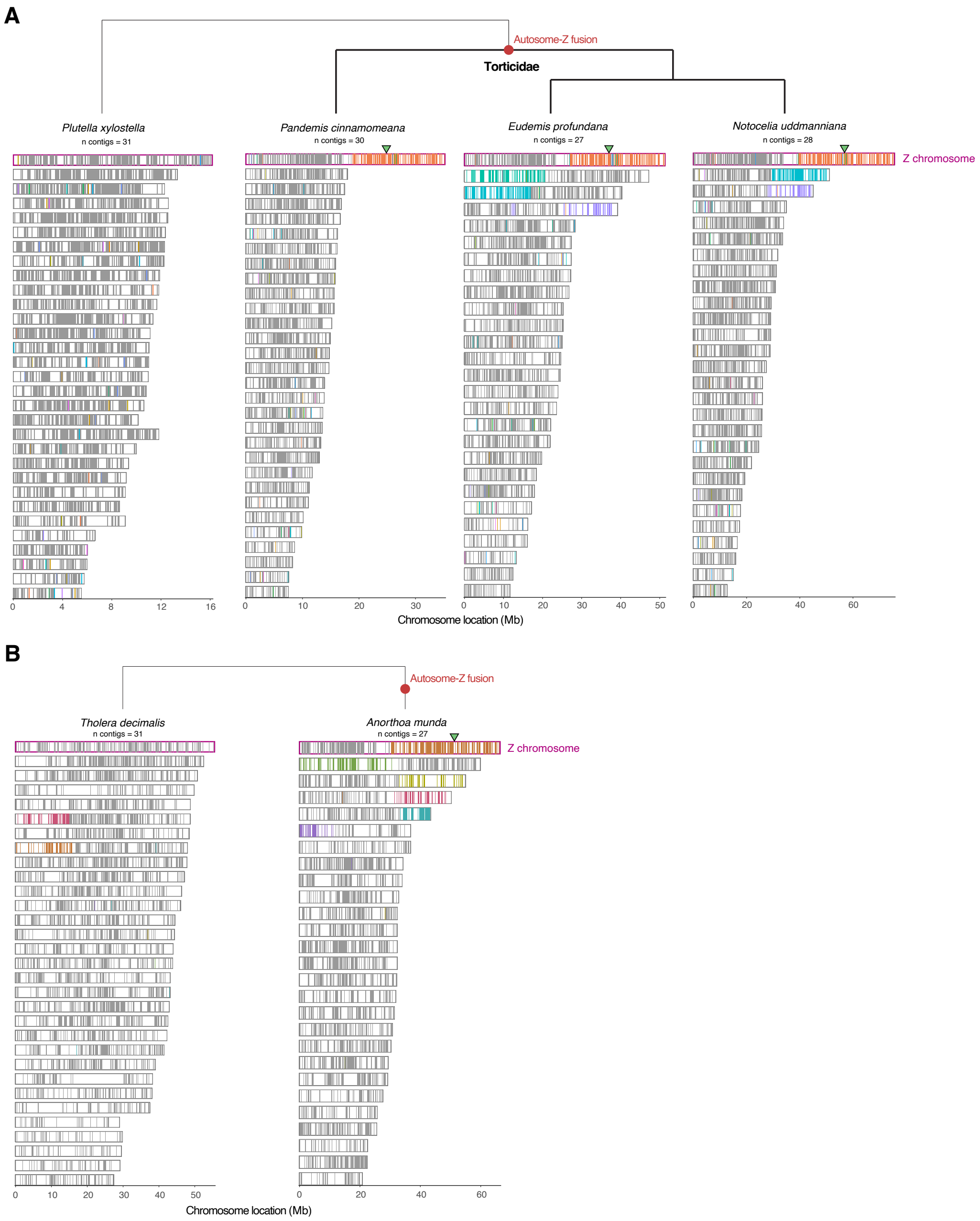

Supplemental Figure S5: Autosome-Z chromosome fusion events in Tortricidae and *Anorthoa munda*. Orthologous BUSCO genes between outgroup and ingroup species are painted onto each chromosome using lepbuscopainter (`https://github.com/charlottewright/lep_busco_painter`). For each species, each chromosome is shown as a rectangle. A grey bar is given for shared orthologous BUSCO genes in the same position as the outgroup species, and a coloured bar is shown for BUSCO genes in an alternative chromosome location compared to the outgroup. This is shown for **(A)** three representative species in Tortricidae compared to *Plutella xylostella* as the outgroup, and **(B)** *Anorthoa munda* compared to *Tholera decimalis* as the outgroup species. In both **A** and **B**, the top chromosome rectangle represents the Z chromosome in all species (highlighted with a pink box) and shows an autosome-Z chromosome fusion in the ingroup species. Additionally, the location of the newly Z-linked LW opsin genes are labelled with green inverted triangles.

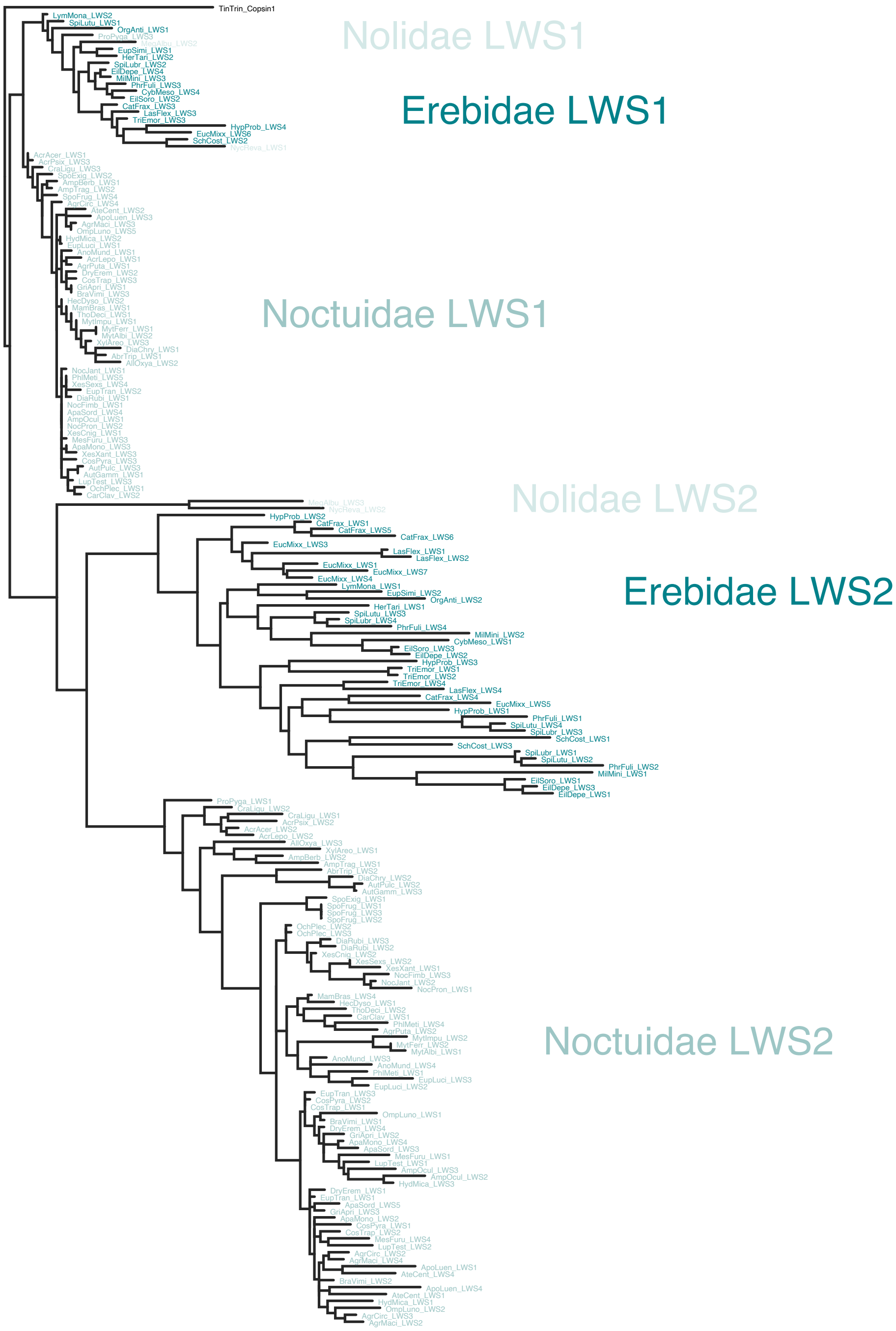

Supplemental Figure S6: Noctuoidea LW opsin gene tree. Gene tree of all LW opsin copies present in species within the superfamily Noctuoidea (Noldiae, Erebidae, and Noctuidae). Each family has a unique colour, and each clade corresponding to an LW gene is labelled. LWS1 represents the parent LW copy in these species, while the LW2 clades represent the ancestrally duplicated LW copy.

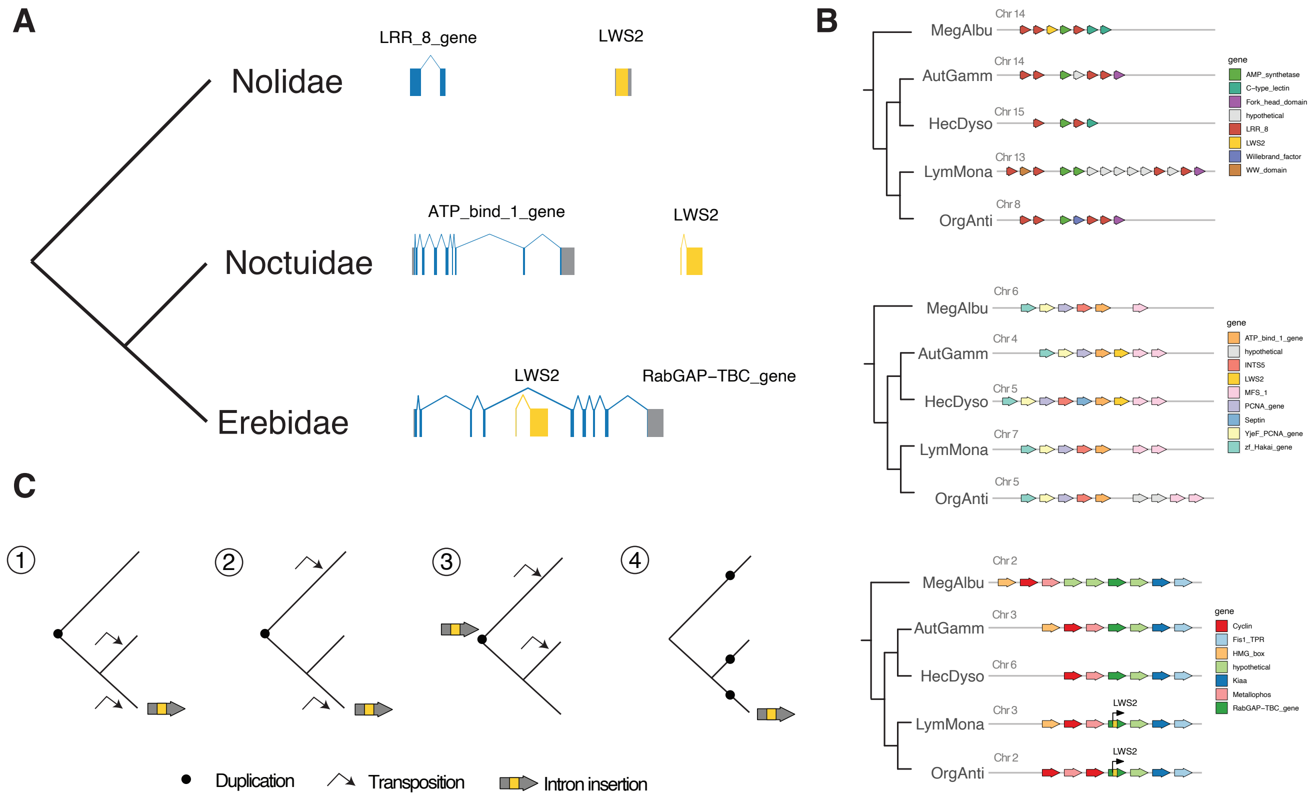

Supplemental Figure S7: Summary of LWS2 paralog genomic locations and duplication history. **(A)** Topology of three families within the Noctuoidea superfamily (left) and the corresponding representative genomic location of the LWS2 copy in each family (right). The LWS2 copy is shown in yellow, while the genes located within the syntenic region in each family is shown in blue and labelled according to the functional domain contained within that gene. **(B)** Family specific syntenic clusters surrounding the LWS2 gene. For each family specific LWS2 syntenic block (top, middle, and bottom), the surrounding genes are shown for *Meganola albula* (Nolidae), *Autographa gamma* (Noctuidae), *Hecatera dysodea* (Noctuidae), *Lymantria monacha* (Erebidae), and *Orgyia antiqua* (Erebidea). The syntenic genes surrounding the LWS2 copy are coloured and the corresponding colour and gene name are given in the legend. **(C)** The hypothetical modes of LWS2 origination and subsequent genome translocations are provided along the topology which corresponds to that in (A).

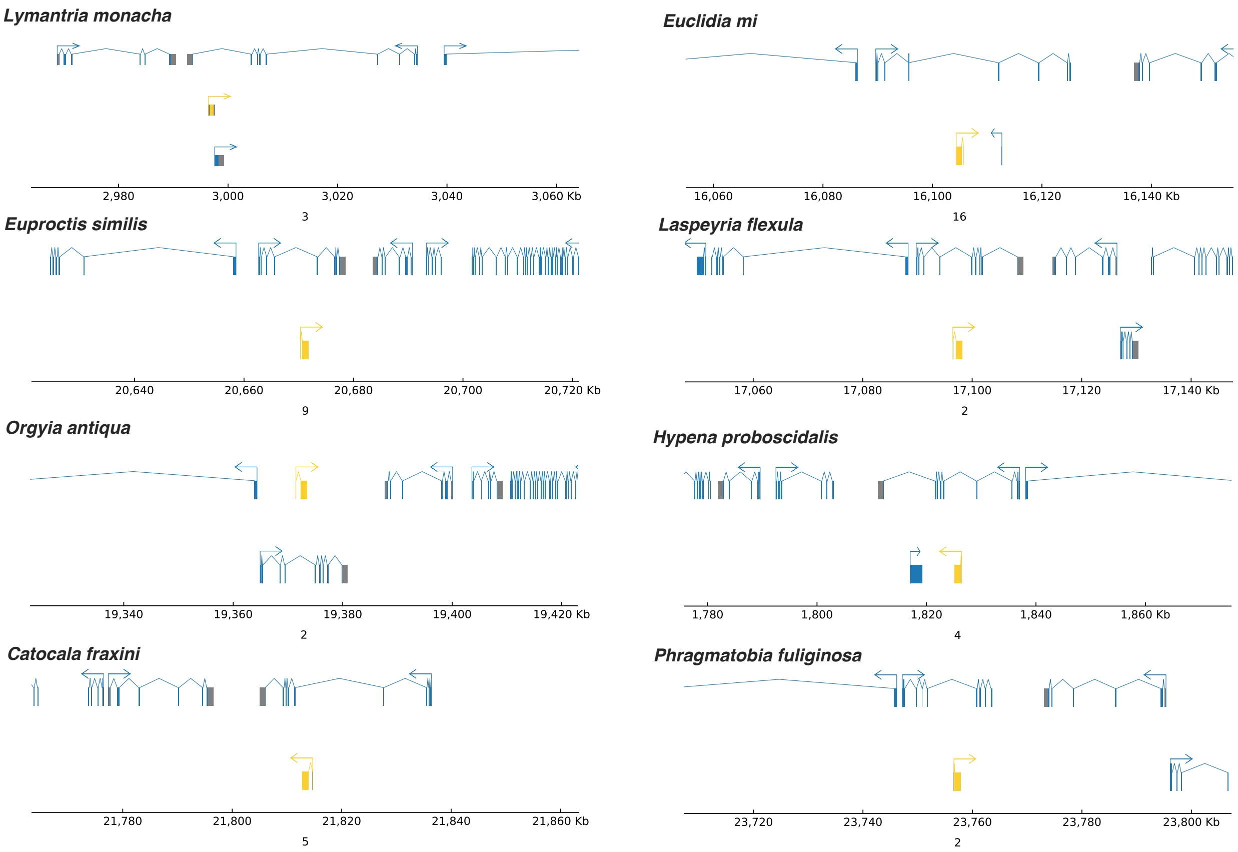

Supplemental Figure S8: Genomic locations of LWS2 paralog in Erebidae species. The location of the LWS2 gene within the intron of another gene, as well as the surrounding syntenic genes, is shown for 8 species from the Erebidae family. In each case the LWS2 gene is shown in yellow, and the surrounding genes are shown in blue. The chromosome and position within the chromosome is given for each one.

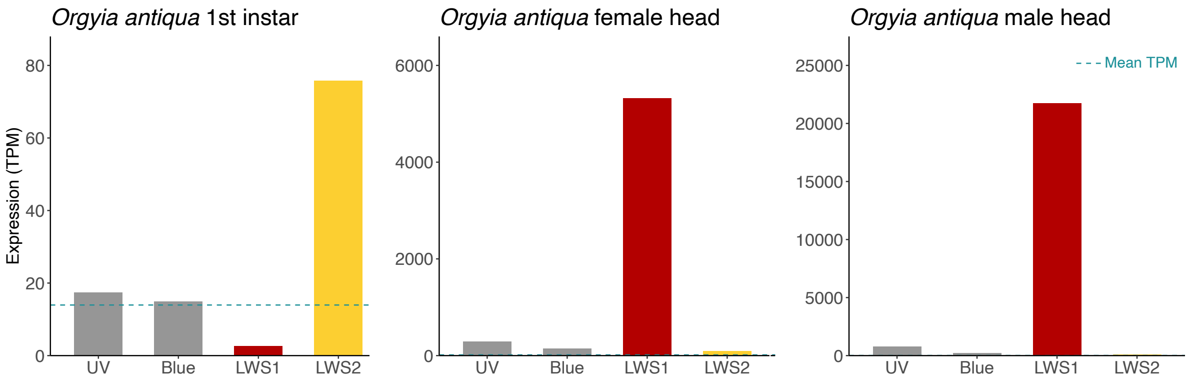

Supplemental Figure S9: TPM values for opsin genes in different lifestages of Vapourer moth (*Orgyia antiqua*). TPM values are given for each of the visual opsin genes (UV, Blue, and LW) in *Orgyia antiqua* first instar larvae (left), female head (middle), and male head (right). In each case, the bar corresponding to the parent LWS1 gene is coloured red and the bar corresponding to the duplicated LWS2 gene is coloured yellow. The average TPM values for each sample is shown by a broken blue line.

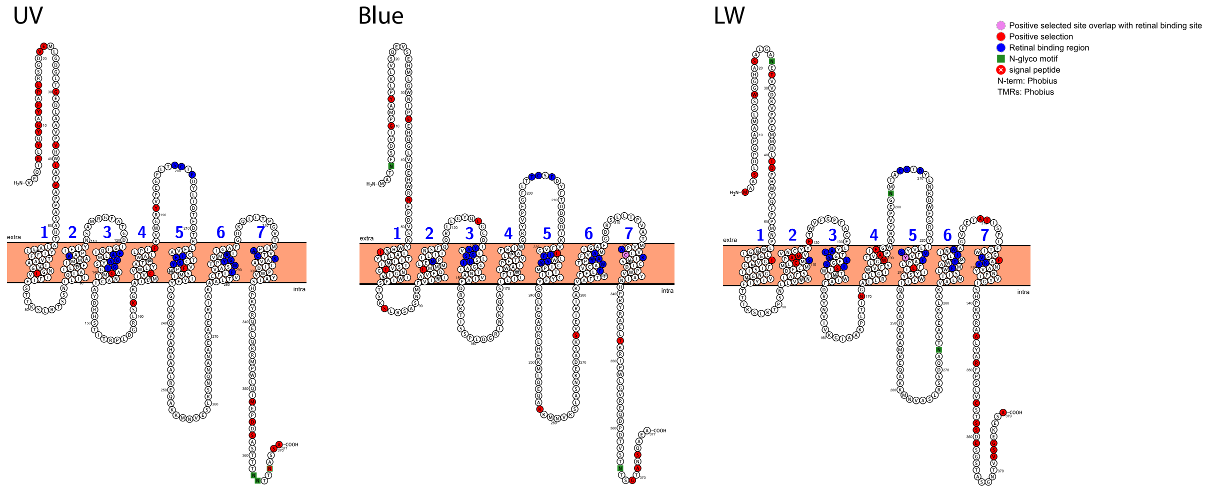

Supplemental Figure S10: Opsin structure with retinal binding sites and overlap with positively selected sites. PHOBIUS transmembrane predictions for each of the three visual opsin genes (UV, Blue, LW) used in the selective pressure analysis. Amino acid sites within 5Å of the chromophore binding pocket (coloured blue) were identified by homology modelling against the crystal structure of Jumping Spider Rhodopsin-1. Sites found to be under positive selection (branch site test) in any of the transition branches from noctural to diurnal species are highlighted by the red colour. Sites where positive selection was found to overlap with retinal binding sites are coloured purple.

Supplementary Tables

Table S1: Species sampled and genome source information.

Species name	Short name	GenBank accession	Project ID
Micropterix aruncella	MicArun	GCA_944548615	PRJEB40665
Neomicropteryx cornuta	NeoCorn	GCA_020383195	PRJNA731916
Incurvaria masculella	IncMasc	GCA_946894095	PRJEB40665
Nematopogon swammerdamellus	NemSwam	GCA_946902875	PRJEB40665
Tinea semifulvella	TinSemi	GCA_910589645	PRJEB40665
Tinea trinotella	TinTrin	GCA_905220615	PRJEB40665
Yponomeuta sedellus	YpoSede	GCA_934045075	PRJEB40665
Plutella xylostella	PluXylo	GCA_932276165	PRJEB40665
Ypsolopha scabrella	YpsScab	GCA_910592155	PRJEB40665
Ypsolopha sequella	YpsSequ	GCA_934047225	PRJEB40665
Pandemis cinnamomeana	PanCinn	GCA_932294345	PRJEB40665
Acleris emargana	AclEmar	GCA_927399475	PRJEB40665
Acleris literana	AclLite	GCA_946894065	PRJEB40665
Acleris sparsana	AclSpar	GCA_923062465	PRJEB40665
Eudemis profundana	EudProf	GCA_947034925	PRJEB40665
Hedya salicella	HedSali	GCA_905404275	PRJEB40665
Apotomis betuletana	ApoBetu	GCA_932273695	PRJEB40665
Apotomis turbidana	ApoTurb	GCA_905147355	PRJEB40665
Cydia splendana	CydSple	GCA_910591565	PRJEB40665
Pammene aurita	PamAuri	GCA_947086415	PRJEB40665
Pammene fasciana	PamFasc	GCA_911728535	PRJEB40665
Notocelia uddmanniana	NotUddm	GCA_905163555	PRJEB40665
Epinotia nisella	EpiNise	GCA_932294315	PRJEB40665
Epinotia bilunana	EpiBilu	GCA_947049275	PRJEB40665
Epinotia demarniana	EpiDema	GCA_945867215	PRJEB40665
Apoda limacodes	ApoLima	GCA_946406115	PRJEB40665
Zygaena filipendulae	ZygFili	GCA_907165275	PRJEB40665
Zeuzera pyrina	ZeuPyri	GCA_907165235	PRJEB40665
Sesia apiformis	SesApif	GCA_914767545	PRJEB40665
Sesia bembeciformis	SesBemb	GCA_943735995	PRJEB40665
Bembecia ichneumoniformis	BemIchn	GCA_910589475	PRJEB40665
Synanthedon andrenaeformis	SynAndr	GCA_936446665	PRJEB40665
Synanthedon myopaeformis	SynMyop	GCA_944738685	PRJEB40665
Synanthedon formicaeformis	SynForm	GCA_945859745	PRJEB40665
Synanthedon vespiformis	SynVesp	GCA_918317495	PRJEB40665
Emmelina monodactyla	EmmMono	GCA_916618145	PRJEB40665
Marasmarcha lunaedactyla	MarLuna	GCA_923062675	PRJEB40665
Stenoptilia bipunctidactyla	SteBipu	GCA_944452665	PRJEB40665
Papilio machaon	PapMach	GCA_912999745	PRJEB40665
Erynnis tages	EryTage	GCA_905147235	PRJEB40665
Pyrgus malvae	PyrMalv	GCA_911387765	PRJEB40665

Carterocephalus palaemon	CarPala	GCA_944567765	PRJEB40665
Thymelicus sylvestris	ThySylv	GCA_911387775	PRJEB40665
Hesperia comma	HesComm	GCA_905404135	PRJEB40665
Ochlodes sylvanus	OchSylv	GCA_905404295	PRJEB40665
Leptidea sinapis	LepSina	GCA_905404315	PRJEB40665
Colias croceus	ColCroc	GCA_905220415	PRJEB40665
Anthocharis cardamines	AntCard	GCA_905404175	PRJEB40665
Aporia crataegi	ApoCrat	GCA_912999735	PRJEB40665
Pieris rapae	PieRapa	GCA_905147795	PRJEB40665
Pieris brassicae	PieBras	GCA_905147105	PRJEB40665
Pieris napi	PieNapi	GCA_905231885	PRJEB40665
Lycaena phlaeas	LycPhla	GCA_905333005	PRJEB40665
Celastrina argiolus	CelArgi	GCA_905187575	PRJEB40665
Glaucopsyche alexis	GlaAlex	GCA_905404095	PRJEB40665
Plebejus argus	PleArgu	GCA_905404155	PRJEB40665
Cyaniris semiargus	CyaSemi	GCA_905187585	PRJEB40665
Aricia agestis	AriAges	GCA_944452695	PRJEB40665
Aricia artaxerxes	AriArta	GCA_937612035	PRJEB40665
Polyommatus icarus	PolIcar	GCA_937595015	PRJEB40665
Lysandra bellargus	LysBell	GCA_905333045	PRJEB40665
Lysandra coridon	LysCori	GCA_905220515	PRJEB40665
Bicyclus anynana	BicAnyn	GCA_947172395	PRJEB40665
Lasiommata megera	LasMege	GCA_928268935	PRJEB40665
Pararge aegeria	ParAege	GCA_905163445	PRJEB40665
Hipparchia semele	HipSeme	GCA_933228805	PRJEB40665
Melanargia galathea	MelGala	GCA_920104075	PRJEB40665
Aphantopus hyperantus	AphHype	GCA_902806685	PRJEB40665
Maniola jurtina	ManJurt	GCA_905333055	PRJEB40665
Erebia aethiops	EreAeth	GCA_923060345	PRJEB40665
Erebia ligea	EreLige	GCA_917051295	PRJEB40665
Limenitis camilla	LimCami	GCA_905147385	PRJEB40665
Boloria selene	BolSele	GCA_905231865	PRJEB40665
Fabriciana adippe	FabAdip	GCA_905404265	PRJEB40665
Mellicta athalia	MelAtha	GCA_905220545	PRJEB40665
Melitaea cinxia	MelCinx	GCA_905220565	PRJEB40665
Vanessa atalanta	VanAtal	GCA_905147765	PRJEB40665
Vanessa cardui	VanCard	GCA_905220365	PRJEB40665
Nymphalis polychloros	NymPoly	GCA_905220585	PRJEB40665
Inachis io	AglIo	GCA_905147045	PRJEB40665
Nymphalis urticae	AglUrti	GCA_905147175	PRJEB40665
Agonopterix subpropinquella	AgoSubp	GCA_922987775	PRJEB40665
Carcina quercana	CarQuer	GCA_910589575	PRJEB40665
Hyposmocoma kahamanoa	HypKaha	GCA_003589595	PRJNA488386
Coleophora flavipennella	ColFlav	GCA_947284805	PRJEB40665
Esperia sulphurella	EspSulp	GCA_947086405	PRJEB40665
Blastobasis adustella	BlaAdus	GCA_907269095	PRJEB40665
Blastobasis lacticolella	BlaLact	GCA_905147135	PRJEB40665

Acrobasis suavella	AcrSuav	GCA_943193695	PRJEB40665
Apomyelois bistriatella	ApoBist	GCA_947044815	PRJEB40665
Endotricha flammealis	EndFlam	GCA_905163395	PRJEB40665
Hypsopygia costalis	HypCost	GCA_937001555	PRJEB40665
Acentria ephemerella	AceEphe	GCA_943193645	PRJEB40665
Parapoynx stratiotata	ParStra	GCA_910589355	PRJEB40665
Calamotropha paludella	CalPalu	GCA_927399485	PRJEB40665
Chrysoteuchia culmella	ChrCulm	GCA_910589605	PRJEB40665
Agriphila geniculea	AgrGeni	GCA_943789525	PRJEB40665
Agriphila tristella	AgrTris	GCA_928269145	PRJEB40665
Drepana falcataria	DreFalc	GCA_945859725	PRJEB40665
Watsonalla binaria	WatBina	GCA_929442735	PRJEB40665
Habrosyne pyritoides	HabPyri	GCA_907165245	PRJEB40665
Thyatira batis	ThyBati	GCA_905147785	PRJEB40665
Saturnia pavonia	SatPavo	GCA_947532125	PRJEB40665
Deilephila porcellus	DeiPorc	GCA_905220455	PRJEB40665
Hemaris fuciformis	HemFuci	GCA_907164795	PRJEB40665
Laothoe populi	LaoPopu	GCA_905220505	PRJEB40665
Mimas tiliae	MimTili	GCA_905332985	PRJEB40665
Ligdia adustata	LigAdus	GCA_947049295	PRJEB40665
Macaria notata	MacNota	GCA_927399415	PRJEB40665
Biston betularia	BisBetu	GCA_905404145	PRJEB40665
Erannis defoliaria	EraDefo	GCA_905404285	PRJEB40665
Peribatodes rhomboidaria	PerRhom	GCA_911728515	PRJEB40665
Agriopis aurantiaria	AgrAura	GCA_914767915	PRJEB40665
Agriopis marginaria	AgrMarg	GCA_932305915	PRJEB40665
Apeira syringaria	ApeSyri	GCA_934044485	PRJEB40665
Selenia dentaria	SelDent	GCA_917880725	PRJEB40665
Alsophila aescularia	AlsAesc	GCA_946251855	PRJEB40665
Campaea margaritaria	CamMarg	GCA_912999815	PRJEB40665
Hylaea fasciaria	HylFasc	GCA_905147375	PRJEB40665
Opisthograptis luteolata	OpiLute	GCA_931315375	PRJEB40665
Crocallis elinguaria	CroElin	GCA_907269065	PRJEB40665
Ennomos fuscantarius	EnnFusc	GCA_905220475	PRJEB40665
Ennomos quercinarius	EnnQuer	GCA_910589525	PRJEB40665
Idaea aversata	IdaAver	GCA_907269075	PRJEB40665
Aplocera efformata	AplEffo	GCA_921293045	PRJEB40665
Lobophora halterata	LobHalt	GCA_932526365	PRJEB40665
Gymnoscelis rufifasciata	GymRufi	GCA_929108375	PRJEB40665
Eupithecia centaureata	EupCent	GCA_944547425	PRJEB40665
Eupithecia abbreviata	EupAbbr	GCA_943735965	PRJEB40665
Eupithecia dodoneata	EupDodo	GCA_947044415	PRJEB40665
Eupithecia exiguata	EupExig	GCA_947086465	PRJEB40665
Eupithecia vulgata	EupVulg	GCA_946478455	PRJEB40665
Operophtera brumata	OpeBrum	GCA_932527175	PRJEB40665
Philereme vetulata	PhiVetu	GCA_918857605	PRJEB40665
Hydriomena furcata	HydFurc	GCA_912999785	PRJEB40665

Xanthorhoe spadicearia	XanSpad	GCA_947086425	PRJEB40665
Ecliptopera silaceata	EclSila	GCA_932527185	PRJEB40665
Eulithis prunata	EulPrun	GCA_918843925	PRJEB40665
Electrophaes corylata	EleCory	GCA_947095575	PRJEB40665
Chloroclysta siterata	ChlSite	GCA_932294275	PRJEB40665
Thera britannica	TheBrit	GCA_939531255	PRJEB40665
Clostera curtula	CloCurt	GCA_905475355	PRJEB40665
Furcula furcula	FurFurc	GCA_911728495	PRJEB40665
Phalera bucephala	PhaBuce	GCA_905147815	PRJEB40665
Pheosia gnoma	PheGnom	GCA_905404115	PRJEB40665
Pheosia tremula	PheTrem	GCA_905333125	PRJEB40665
Ptilodon capucinus	PtiCapu	GCA_914767695	PRJEB40665
Notodonta dromedarius	NotDrom	GCA_905147325	PRJEB40665
Notodonta ziczac	NotZicz	GCA_918843915	PRJEB40665
Meganola albula	MegAlbu	GCA_936450015	PRJEB40665
Nycteola revayana	NycReva	GCA_947037095	PRJEB40665
Lymantria monacha	LymMona	GCA_905163515	PRJEB40665
Euproctis similis	EupSimi	GCA_905147225	PRJEB40665
Orgyia antiqua	OrgAnti	GCA_916999025	PRJEB40665
Catocala fraxini	CatFrax	GCA_930367265	PRJEB40665
Euclidia mi	EucMi	GCA_944739405	PRJEB40665
Schrankia costaestrigalis	SchCost	GCA_905475405	PRJEB40665
Laspeyria flexula	LasFlex	GCA_905147015	PRJEB40665
Trisateles emortualis	TriEmor	GCA_947095525	PRJEB40665
Hypena proboscidalis	HypProb	GCA_905147285	PRJEB40665
Herminia tarsipennalis	HerTars	GCA_945859575	PRJEB40665
Phragmatobia fuliginosa	PhrFuli	GCA_932526445	PRJEB40665
Spilosoma lubricipeda	SpiLubr	GCA_905220595	PRJEB40665
Spilarctia lutea	SpiLute	GCA_916048165	PRJEB40665
Miltochrista miniata	MilMini	GCA_933228765	PRJEB40665
Cybosia mesomella	CybMeso	GCA_946251805	PRJEB40665
Eilema depressum	EilDepr	GCA_914767945	PRJEB40665
Eilema sororculum	EilSoro	GCA_914829495	PRJEB40665
Abrostola tripartita	AbrTrip	GCA_946251915	PRJEB40665
Diachrysia chrysitis	DiaChry	GCA_932294365	PRJEB40665
Autographa gamma	AutGamm	GCA_905146925	PRJEB40665
Autographa pulchrina	AutPulc	GCA_905475315	PRJEB40665
Protodeltote pygarga	ProPyga	GCA_936450705	PRJEB40665
Allophyes oxyacanthae	AllOxya	GCA_932294325	PRJEB40665
Craniophora ligustri	CraLigu	GCA_905163465	PRJEB40665
Acronicta psi	AcrPsi	GCA_946251955	PRJEB40665
Acronicta aceris	AcrAcer	GCA_910591435	PRJEB40665
Acronicta leporina	AcrLepo	GCA_947256265	PRJEB40665
Xylocampa areola	XylAreo	GCA_935421205	PRJEB40665
Amphipyra berbera	AmpBerb	GCA_910594945	PRJEB40665
Amphipyra tragopoginis	AmpTrag	GCA_905220435	PRJEB40665
Spodoptera exigua	SpoExig	GCA_902829305	PRJEB36598

Spodoptera frugiperda	SpoFrug	GCA_011064685	PRJNA590312
Caradrina clavipalpis	CarClav	GCA_932526535	PRJEB40665
Anorthoa munda	AnoMund	GCA_945859665	PRJEB40665
Tholera decimalis	ThoDeci	GCA_943138885	PRJEB40665
Hecatera dysodea	HecDyso	GCA_905332915	PRJEB40665
Mamestra brassicae	MamBras	GCA_905163435	PRJEB40665
Mythimna impura	MytImpu	GCA_905147345	PRJEB40665
Mythimna albipuncta	MytAlbi	GCA_929112965	PRJEB40665
Mythimna ferrago	MytFerr	GCA_910589285	PRJEB40665
Agrotis puta	AgrPuta	GCA_943136025	PRJEB40665
Ochropleura plecta	OchPlec	GCA_905475445	PRJEB40665
Diarsia rubi	DiaRubi	GCA_932274075	PRJEB40665
Noctua pronuba	NocPron	GCA_905220335	PRJEB40665
Noctua fimbriata	NocFimb	GCA_905163415	PRJEB40665
Noctua janthe	NocJant	GCA_910589295	PRJEB40665
Xestia c-nigrum	XesCnig	GCA_916618015	PRJEB40665
Xestia sexstrigata	XesSexs	GCA_941918905	PRJEB40665
Xestia xanthographa	XesXant	GCA_905147715	PRJEB40665
Euplexia lucipara	EupLuci	GCA_921972225	PRJEB40665
Phlogophora meticulosa	PhlMeti	GCA_905147745	PRJEB40665
Atethmia centrago	AteCent	GCA_905333075	PRJEB40665
Cosmia pyralina	CosPyra	GCA_946251885	PRJEB40665
Cosmia trapezina	CosTrap	GCA_905163495	PRJEB40665
Eupsilia transversa	EupTran	GCA_914767815	PRJEB40665
Omphaloscelis lunosa	OmpLuno	GCA_916610215	PRJEB40665
Agrochola circellaris	AgrCirc	GCA_914767755	PRJEB40665
Agrochola macilenta	AgrMaci	GCA_916701695	PRJEB40665
Brachylomia viminalis	BraVimi	GCA_937001585	PRJEB40665
Dryobotodes eremita	DryErem	GCA_917490735	PRJEB40665
Griposia aprilina	GriApri	GCA_916610205	PRJEB40665
Aporophyla lueneburgensis	ApoLuen	GCA_932294355	PRJEB40665
Apamea monoglypha	ApaMono	GCA_911387795	PRJEB40665
Apamea sordens	ApaSord	GCA_945859715	PRJEB40665
Amphipoea oculea	AmpOcul	GCA_945859645	PRJEB40665
Hydraecia micacea	HydMica	GCA_914767645	PRJEB40665
Luperina testacea	LupTest	GCA_927399505	PRJEB40665
Mesoligia furuncula	MesFuru	GCA_916614155	PRJEB40665
